# Self-worth and bonding emotions are related to well-being in health-care providers: a cross-sectional study

**DOI:** 10.1186/s12909-021-02731-7

**Published:** 2021-05-21

**Authors:** Sonja Weilenmann, Ulrich Schnyder, Nina Keller, Claudio Corda, Tobias R. Spiller, Fabio Brugger, Brian Parkinson, Roland von Knel, Monique C. Pfaltz

**Affiliations:** 1 Department of Consultation-Liaison Psychiatry and Psychosomatic Medicine, University Hospital Zurich, Haldenbachstrasse 18, CH-8091 Zurich, Switzerland; 2 University of Zurich, Zurich, Switzerland; 3 Department of Experimental Psychology, University of Oxford, Oxford, UK

**Keywords:** Emotions, Basic needs, Self-worth, Bonding, Eudaimonic well-being, Hedonic well-being, Burnout, Physicians, Nurses, Psychotherapists

## Abstract

**Background:**

Interacting with patients can elicit a myriad of emotions in health-care providers. This may result in satisfaction or put providers at risk for stress-related conditions such as burnout. The present study attempted to identify emotions that promote provider well-being. Following eudaimonic models of well-being, we tested whether certain types of emotions that reflect fulfilment of basic needs (self-worth, bonding with patients) rather than positive emotions in general (as suggested by hedonic models) are linked to well-being. Specifically, we hypothesized that well-being is associated with positive emotions directed at the self, which reflect self-worth, and positive as well as negative emotions (e.g., worry) directed at the patient, which reflect bonding. However, we expected positive emotions directed at an object/situation (e.g., curiosity for a treatment) to be unrelated to well-being, because they do not reflect fulfilment of basic needs.

**Methods:**

Fifty eight physicians, nurses, and psychotherapists participated in the study. First, in qualitative interviews, they reported their emotions directed at the self, the patient, or an object/situation during distressing interactions with patients. These emotions were categorised into positive emotions directed towards the self, the patient, and an object/situation, and negative emotions directed towards the patient that reflect bonding. Second, providers completed questionnaires to assess their hedonic and eudaimonic well-being. The well-being scores of providers who did and did not experience these emotions were compared.

**Results:**

Providers who experienced positive emotions directed towards the self or the patient had higher well-being than those who did not. Moreover, for the first time, we found evidence for higher well-being in providers reporting negative patient-directed emotions during distressing interactions. There was no difference between providers who did and did not experience positive object/situation-directed emotions.

**Conclusions:**

These findings may point towards the importance of eudaimonic emotions rather than just positive emotions in interactions with patients. Emotions such as contentment with oneself, joy for the patients improvement, and, notably, grief or worry for the patient may build a sense of self-worth and strengthen bonding with the patient. This may explain their association with provider well-being.

**Supplementary Information:**

The online version contains supplementary material available at 10.1186/s12909-021-02731-7.

## Background

Caring for patients can elicit various emotions in health-care providers. While some of these emotions can result in positive provider outcomes such as satisfaction or professional fulfilment, others may put providers at risk for stress-related conditions such as burnout. These conditions are highly prevalent in health-care professions [1,7]. For example, studies of physicians, nurses, and psychotherapists show that caring empathically for others may elicit feelings of joy, fulfilment, gratitude, and inspiration [8,13]. By contrast, situations such as treating suffering patients, breaking bad news, being exposed to the traumatic contents of patients histories, and losing patients to death may lead to doubt, grief, shock, and guilt [14,21]. Hence, health-care-related emotions clearly carry the potential for both positive and negative provider outcomes (e.g., satisfaction and burnout) [1, 22,25]. However, no previous study has assessed which of these emotions are associated with provider well-being. To elucidate this association, we look at two existing models of well-being.

According to the widespread hedonic model of well-being, experiencing high levels of positive emotions and low levels of negative emotions enhances well-being [26,28]. Although empirically supported, this hedonic model may not be sufficient in explaining well-being in health-care professionals. For example, are negative emotions such as grieving for patients indeed unfavourable for the provider? Or can they give a sense of meaning? Moreover, is it not also important to consider the target an emotion is directed at [29]? For example, positive emotions directed at oneself (e.g., pride in ones performance) may have a larger effect on well-being than positive emotions directed at an object (e.g., curiosity about a disease). Taken together, it may be too simplistic to view positive emotions as favourable and negative emotions as unfavourable to well-being irrespective of their content and directedness. Therefore, more refined models are needed.

Expanding hedonic theories, Tamir and colleagues have highlighted the importance for well-being of emotions that serve eudaimonic purposes [30, 31]. In eudaimonic models of well-being, well-being results from realising ones potential rather than from experiencing positive emotions. Realising ones potential, also called positive functioning or flourishing, has been characterised by varying constructs such as environmental mastery, meaning in life, and personal growth [32,36]. Moreover, at the core of several of the eudaimonic well-being models are two constructs from basic needs theories [37, 38], which also reflect positive functioning [32,36]: The first concerns the need to feel valuable, lovable, and competent, here referred to as need for self-worth. The second concerns the need to be close and connected to others, here referred to as need for bonding [39]. As suggested by Tamir and colleagues, emotions that reflect basic needs serve eudaimonic purposes and may therefore be associated with well-being [30, 31].

No research has yet defined the kinds of emotions that reflect the fulfilment of these two basic needs. However, Fischer and Manstead [40] have made important contributions to our understanding of the social functions of emotions. According to these authors, emotions can serve the goal of affiliation or social distancing. Prototypical examples for emotions that enhance closeness or affiliation between individuals are love and happiness in terms of sharing positive experiences, and sadness when seeking support. In contrast, anger, contempt, or fear of another person are emotions that foster social distance [40].

The concept of Fischer and Manstead could be applied to the health-care context. Emotions of affection and joy directed towards the patient may serve affiliative functions and thus reflect fulfilment of the need for bonding. Fischer and Manstead argue that negative emotions can also serve affiliative functions [40]. We therefore propose that patient-directed negative emotions of fear for the patient (e.g., worrying) or sadness for the patient (e.g., grieving) also reflect social bonding. In contrast, emotions such as anger, dislike, and disappointment directed towards the patient may instead serve social distancing functions.

The other need proposed in eudaimonic models of well-being is the need for self-worth. It is known that emotions may serve a self-evaluative function [41, 42]. For example, people who attribute success to their own abilities or behaviours experience pride (see also [43]). Hence, pride is a positive self-evaluative emotion. In contrast, people who attribute failure to their inability or wrong behaviour experience guilt. Guilt is thus a negative self-evaluative emotion [42, 43]. Based on these considerations, we propose that positive emotions of affection and joy directed towards the self (e.g., liking oneself as a person, satisfaction or pride with ones performance) are positive self-evaluations. Therefore, these emotions promote high self-worth. In contrast, negative self-directed emotions (e.g., anger or doubt about ones performance) are negative self-evaluations and reflect low self-worth.

The eudaimonic model extends the hedonic model in one particularly important way. Unlike in hedonic models of well-being, emotions that serve eudaimonic purposes are thought to foster well-being irrespective of their valence [30, 31]. Hence, positive emotions directed at oneself or the patient will likely be important to well-being not merely because they are positive, but also because they reflect fulfilment of basic needs. In contrast, positive emotions directed at an object or situation (e.g., treatment, disease) may be less important and play little role in enhancing well-being, even though they are positive. Moreover, if they reflect bonding with the patient, negative emotions directed at the patient could be favourable to well-being, despite their felt unpleasantness.

To verify these assumptions, we aimed to assess whether positive self-directed emotions (self-worth emotions), positive patient-directed emotions (positive bonding emotions), and certain negative patient-directed emotions (i.e., fear or sadness for the patient; negative bonding emotions) are positively related to well-being in health-care providers. By contrast, we did not expect positive object-directed emotions to be strong predictors of well-being.

## Methods

Since no previous study has investigated the different kinds of emotions that arise during patient-provider interactions, there was no suitable instrument to assess emotions within this setting. Therefore, data collection consisted of two steps. First, we conducted a series of in-depth interviews with health-care providers, asking them to report on emotions they experience during distressing interactions with their patients. We documented their (positive and negative) emotions directed at the self, the patient, or an object/situation. Second, directly after the interview, providers completed questionnaires to assess their well-being. Finally, we tested whether the positive self-directed emotions, positive patient-directed emotions, negative patient-directed, and positive object-directed emotions were related to well-being as predicted.

### Participants

A convenience sample of 58 health-care providers from the German-speaking part of Switzerland were recruited from hospitals, private practices, and home-care services. Sample characteristics are detailed in Table1.

**Table 1:** Sample characteristics

		***n***	
Total		58	
Women		41	
Men		17	
Physicians			
Total		24	
Inpatient		8	
Outpatient		16	
Nurses			
Total		17	
Inpatient		14	
Outpatient		3	
Psychological Psychotherapists			
Total		17	
Inpatient		9	
Outpatient		8	
	***M***	***SD***	***Range***
Age (years)	42.48	12.81	2374
Percentage of work (%)	75.95	25.76	20100
Professional experience (years)	15.14	11.335	0.540
Eudaimonic well-being	5.81	.73	3.887
Hedonic well-being	3.77	.82	1.675

### Measures

#### Emotions

Emotions were assessed using in-depth interviews. Interviews focused on recent interactions with patients that providers had perceived as distressing, because distressing interactions carry the potential for bringing both positive and negative well-being outcomes for the provider, and are the ones that have repeatedly been indicated to play a crucial role in provider well-being and burnout [29]. Most of the recalled interactions were distressing either because they involved patients who were severely ill (e.g., in palliative care) or difficult to treat for some other reason (e.g., certain personality traits). Most of the interactions selected by interviewees had occured during the previous month, with few having happened between one and several months (but less than a year) ago.

Interviews were conducted by SW (psychologist) and NK (psychology student trained by SW). Together with the interviewer, interviewees explored their emotions and emotion regulation (reported elsewhere) with regard to this interaction. To this end, a manual, which we had developed for this study, was used (see Additional file 1). Specifically, interviewees were asked the following main open-ended question: Which emotions did you experience while interacting with the patient? Interviewees responses were followed up by more specific questions asking for the provider-perceived directedness of each emotion (e.g., self-, patient-, and object/situation-directed [29]; some emotions were directed at other persons present during the interaction, which was outside the scope of the present study). Each emotion together with its directedness as stated by the interviewee was collected in an interview protocol sheet (see Additional file 1).

For each kind of directedness (self, patient, object/situation) separately, the emotions collected during the interviews were categorised using the classification developed by Shaver and colleagues [44]. This classification categorises various examples of emotions into the higher order categories *affection* and *joy* (positive emotions), and *anger*, *sadness*, and *fear* (negative emotions). SW and NK assigned the emotions from the interviewees to the higher order category that included the same or matching examples of emotions. The resulting categorisation of emotions was reviewed by US, BP, and MP, and is displayed in Table2. A description of providers emotions can be found in the results section.

**Table 2:** Providers emotions in provider-patient interactions

**Emotions directed at the providers themselves**	**P (*n*)**	**N (*n*)**	**T (*n*)**	**Total (*n*)**
**Affection**				0
Affection	0	0	0	0
**Joy**				27
Joy	1	0	0	1
Contentment	3	2	4	9
Pride	0	3	3	6
Optimism, confidence, hope	1	3	3	7
Relief	0	1	3	4
**Anger**				7
Irritation	2	0	2	4
Exasperation, frustration	1	2	0	3
**Sadness**				23
Sadness, consternation	2	1	1	4
Disappointment	3	0	0	3
Shame, guilt	4	3	3	10
Neglect, insult	5	0	0	5
Sympathy	1	0	0	1
**Fear**				39
Nervousness, fear of failure, helplessness, doubt, incompetence	15	11	13	39
**Emotions directed at the patients**	**P (*n*)**	**N (*n*)**	**T (*n*)**	**Total (*n*)**
**Affection**				25
Affection, compassion, liking, benevolence	10	5	10	25
**Joy**				24
Joy	1	2	2	5
Zest, curiosity, interest	2	0	2	4
Contentment	0	0	2	2
Optimism, confidence	3	1	2	6
Amazement	2	1	2	5
Relief	0	2	0	2
**Anger**				34
Irritation, annoyance, incomprehension	8	7	4	19
Exasperation, frustration	1	4	1	6
Anger	6	2	4	12
Disgust, disliking	4	3	0	7
**Sadness**				26
Sadness	2	4	3	9
Sympathy	5	2	1	8
Disappointment	5	3	2	10
**Fear**				13
Nervousness (for the patient), worry, fear for the patient	3	1	7	11
Nervousness (about the patient), being afraid of the patient	2	1	0	3
**Emotions directed at an object/situation**	**P (*n*)**	**N (*n*)**	**T (*n*)**	**Total (*n*)**
**Affection**				0
Affection	0	0	0	0
**Joy**				29
Zest, curiosity, interest	2	2	1	5
Contentment	1	2	2	5
Optimism, confidence, security	3	0	2	5
Relief	9	7	4	20
**Anger**				22
Irritation, impatience	8	2	2	12
Exasperation, frustration	5	4	2	11
Anger	1	1	2	4
**Sadness**				25
Sadness, powerlessness, hopelessness	9	4	4	17
Disappointment, dismay	7	2	2	11
**Fear**				27
Nervousness, fear, insecurity, tenseness	13	8	6	27

*Note*. Numbers of physicians (P), nurses (N), psychotherapists (T) and interviewees (P, N, and T combined) who reported the respective self-, patient-, and object/situation-directed emotions. Shavers [44] subcategory names are in the first place, supplemented with further examples of the most frequently reported emotions by interviewees. The subcategory name cheerfulness was replaced by joy, as none of the interviewees reported cheerfulness. Similarly, ragewas replaced by angerand enthrallmentby amazement

For the statistical analysis of the relationship between well-being and emotions serving eudaimonic purposes, we defined the categories of emotions that serve eudaimonic purposes, i.e., positive self-directed emotions, positive patient-directed emotions, and negative patient-directed emotions that reflect bonding (see Table3). Henceforth, we call these emotions *eudaimonic emotions*.

**Table 3:** Eudaimonic emotions

Emotions	Eudaimonic purpose
Self-directed positive emotions, i.e., emotions in the categories affection and joy	*Self-worth*
Patient-directed positive emotions, i.e., emotions in the categories affection and joy	*Bonding with the patient*
Patient-directed negative emotions that reflect bonding, i.e., emotions in the categories sadness and fear	*Bonding with the patient*

*Note.* Not all patient-directed sadness and fear emotions are likely to reflect bonding. To demarcate emotions that reflect bonding with the patient from those that reflect distance from the patient, we followed the theoretical considerations from Fischer & Manstead [40] and excluded disappointment among the sadness emotions and being afraid of the patient among the fear emotions from our analysis

Not all interviewees had reported emotions from all types of eudaimonic emotions listed in Table 3. Therefore, we decided to divide our sample into two subsamples for each type of eudaimonic emotions: In each case, the first subsample comprised interviewees who had not experienced the type of eudaimonic emotions in question (e.g., interviewees who did not experience self-directed positive emotions), the second subsample contained interviewees having experienced these emotions (e.g., interviewees who did experience self-directed positive emotions).

#### Well-being

After the interview, providers completed pen-and-paper questionnaires to retrospectively assess their state well-being directly after the interaction they had selected. We measured eudaimonic well-being using the validated German version of the 8-item *Flourishing Scale* (FS; 7-point Likert scale, 1=strongly disagree to 7=strongly agree), which includes items assessing meaning in life, positive relations, mastery, and related concepts [45, 46]. In addition, we measured hedonic well-being using the validated German version of the subjective well-being scales from the *Comprehensive Inventory of Thriving* (CIT; 5-point Likert scale, 1=strongly disagree to 5=strongly agree) [47, 48]. These scales assess positive affect, negative affect, and life satisfaction. Following usual practice, ratings on FS items were summed together to yield a total score, and ratings on CIT items were averaged to yield mean scores (with higher scores indicating higher well-being in both cases). When responding to the well-being items, providers were asked to retrospectively rate their state immediately after the interaction they had selected for the interview. Items of the CIT had to be slightly adapted (i.e., most of the time in six items was dropped) for present purposes.

### Statistical analysis

Statistical analyses were run using R, Version 3.5.3. We compared the hedonic and eudaimonic well-being scores between providers who had (group 2) and who had not (group 1) experienced eudaimonic emotions for each subdivision (self-directed positive emotions, patient-directed positive emotions, patient-directed negative emotions) separately. Furthermore, since we argued that object-directed positive emotions play little role in enhancing well-being, we compared the hedonic and eudaimonic well-being scores for providers who had (group 2) and had not (group 1) experienced positive object-directed emotions. Because well-being was not normally distributed, we used a Mann-Whitney U test to compare the groups.

## Results

### Description of emotions in provider-patient interactions

Table 2 depicts all self-, patient-, and object/situation-directed emotions sorted according to the emotion categories by Shaver and colleagues [44]. The most frequently reported self-directed emotions belonged to the category of nervousness (e.g., fear of failure), but providers also reported being confident in themselves and experiencing contentment about their performance. Among the most frequently reported emotions directed at the patient were emotions of affection for the patient (e.g., liking), anger about the patient (e.g., annoyance), and sadness (i.e., sympathy). Regarding object/situation-directed emotions, nervousness (e.g., tenseness about how a situation would develop), relief (e.g., when a problem was resolved), and sadness (e.g., powerlessness in the face of a disease) were most frequently experienced.

### Relationship between eudaimonic emotions and well-being

Table4 depicts results from the group comparisons. Interviewees who had experienced self-directed positive emotions reported a higher eudaimonic (*U*=215.00, *p*=.007, *r*=.43) and hedonic well-being (*U*=237.00, *p*=.019, *r*=.38) than those who had not. Patient-directed positive emotions were only related to hedonic well-being (*U*=279.50, *p*=.033, *r*=.33). There was no difference in well-being between providers with and without object/situation-directed positive emotions. Furthermore, having experienced negative patient-directed emotions that reflect bonding was associated with both higher eudaimonic (*U*=216.00, *p*=.003, *r*=.46) and hedonic well-being (*U*=239.00, *p*=.009, *r*=.41).

**Table 4:** Relationship between emotions and well-being

	Group 1 (***N***) ***Median***	Group 2 (***N***) ***Median***	***U***	***p***	***r***
**Self-directed positive emotions**	[38]	[20]			
Eudaimonic well-being	5.81	6.25	215.00	.007	*r*=.43
Hedonic well-being	3.78	4.22	237.00	.019	*r*=.38
**Patient-directed positive emotions**	[26]	[32]			
Eudaimonic well-being	5.94	5.94	408.50	n.s.	
Hedonic well-being	3.69	4.11	279.50	.033	*r*=.33
**Object-directed positive emotions**	[29]	[29]			
Eudaimonic well-being	5.88	6.00	398.50	n.s.	
Hedonic well-being	3.88	4.11	324.50	n.s.	
**Patient-directed negative emotions reflecting bonding**	[35]	[23]			
Eudaimonic well-being	5.63	6.13	216.00	.003	*r*=.46
Hedonic well-being	3.78	4.22	239.00	.009	*r*=.41

*Note*. Comparison of well-being between interviewees who had (group 2) and had not (group 1) experienced the respective emotions

## Discussion

In the present study, we set out to identify emotions in health-care interactions that are linked to health-care provider well-being. The widely used hedonic model of well-being postulates that positive emotions are positively associated with well-being and negative emotions negatively so. We found evidence for a more fine-grained understanding of these associations in health-care providers. Rather than positive emotions in general, only positive emotions directed towards the self or the patient seem to be implicated in well-being. Moreover, for the first time, we found evidence that negative patient-directed emotions such as grieving for the patient were positively associated with well-being in distressing interactions. These findings are consistent with our assumptions based on eudaimonic models of well-being [32, 34,36], and may point towards the importance of eudaimonic emotions. In other words, emotions such as contentment with oneself, joy for the patients improvement, and, notably, grief or worry for the patient are related to well-being, arguably because they build a sense of self-worth and strengthen bonding with the patient.

Importantly, these eudaimonic emotions were not only associated with eudaimonic well-being but also with hedonic well-being. This is not surprising given the fact that eudaimonic and hedonic well-being are related to and mutually reinforce one another [35, 36]. Hence, eudaimonic emotions may directly contribute to hedonic well-being or indirectly contribute to hedonic well-being as a consequence of their direct effects on eudaimonic well-being (or both). Moreover, our findings suggest that in health-care providers, even hedonic well-being might not merely be the product of positive emotions, but also other processes (e.g., eudaimonic ones). This is specifically indicated by the absence of any relation between hedonic well-being and positive object/situation-directed emotions and the positive relation between hedonic well-being and negative bonding emotions.

More generally, an important implication of this study is that eudaimonic models may be better suited to identify emotions that are linked to provider well-being than purely hedonic models. Furthermore, it should be highlighted that negative bonding emotions may in fact be beneficial for providers. This finding stands in contrast with literature on empathy-related processes, where sharing suffering with patients is generally considered to be harmful [49, 50]. More research is needed to elucidate the role of negative bonding emotions. For example, there might be an intensity level where bonding emotions turn harmful. Moreover, Tamir and colleagues highlighted the importance of experiencing contextually useful or desired emotions [30, 51]. Hence, in contexts where empathy could disrupt clinical objectivity and performance, it may not be favourable to well-being [49, 52,54]. Consistent with this notion, Decety and colleagues were able to show that physicians downregulate their pain when watching images with body parts in painful conditions [55]. The authors suggest that downregulation is necessary to be able to perform painful medical procedures. Finally, personality traits may play an important role when determining the effects of bonding emotions. Indeed, Tamir and colleagues [30, 31] and Tsai [56] pointed out that only emotional states congruent with an individuals motives and characteristics are related to well-being. Consequently, bonding emotions might only be relevant for providers who value close relationships with their patients.

There are several limitations to the design of the present study. First, results may be affected by recall bias. Evidence demonstrates that memory of emotional episodes is strongly affected by the overall strongest emotion and emotions experienced at the end of a specific episode (see peak-and-end rule [57]). However, this is not necessarily a limitation, because those emotions lingering on for some time might, in our opinion, be more important to well-being than what providers feel at any given moment. Moreover, due to the large time gap, emotions and the subsequent evaluation of ones well-being may to some extent have been retrospectively constructed, which could involve reappraisal processes of the situation based on later encounters with the same patient or other experiences. Also, emotions are subject to many influences, including the effects of emotion regulation. Hence, the reported emotions may not be the unadulterated emotions that were originally felt in the given situation. Taken together, our findings may be more representative of providers beliefs about emotions and their relationship to well-being than of actual emotional processes. Therefore, results must be replicated by using more real-time emotion assessments [58].

Second, recruiting a convenience sample may have introduced a selection bias because providers interested in participating in this study may differ from those who were not. Moreover, due to the small sample size, there was not enough power to test for differences in results with regards to demographic factors (e.g., profession). For the same reason, we were unable to include variables such as personality traits or work characteristics that may have influenced our findings.

Third, the cross-sectional study design prevents from exploring causal and within-person relationships. Clearly, experimental or prospective studies replicating our findings are needed.

## Conclusions

Our study is the first to use a eudaimonic model to understand the relationship between emotions in provider-patient interactions and provider well-being. Findings indicate that experiencing eudaimonic emotions rather than merely enhancing positive emotions and reducing negative ones may be key to well-being. Therefore, this study presents a promising basis for future studies on eudaimonic emotions in particular and on the benefits and costs of health-care interactions in general. By informing education and practice, research along this line may raise awareness of favorable emotional states, stimulate interventions, and ultimately facilitate provider well-being. As provider well-being and good, empathetic patient-provider relationships are associated with quality of care and various patient benefits (e.g. higher treatment adherence) [1, 4, 13, 24, 59,69], experiencing optimal emotional states may translate into improved patient well-being and, ultimately, to more effective health-care systems.

## Supplementary Information

**Additional file 1:**

## Data Availability

The datasets generated and/or analysed during the current study are not publicly available due to the participants not having consented to the publishing of their data but are available from the corresponding author on reasonable request.

## Abbreviations

CIT: Comprehensive Inventory of Thriving
FS: Flourishing Scale
