# Supplementary material for: Self-worth and bonding emotions are related to well-being in health-care providers: a cross-sectional study
Source: BMC Med Educ. 2021 May 21;21:290. doi: 10.1186/s12909-021-02731-7 (PMC8139026; doi:10.1186/s12909-021-02731-7)
Supplement: Supplementary file 1 — Additional file 1: [file 12909_2021_2731_MOESM1_ESM.docx]

**Appendix A**

**Interview manual**

Question 1: Please think of an interaction with a patient that you have experienced

- during the past two weeks (if possible) and
- that you did find distressing.

Please tell me briefly about this interaction.

Emotions

Question 2: Which emotions did you experience while interacting with the patient?

Follow-up question for each emotion if not clear: What was the direction of the emotion (yourself, the patient, something else)?

Instruction to the interviewer: Complete this form for each emotion the provider has experienced (if an emotion has several directions at the same time, list them separately).

| **Emotions:** | **Direction (self, patient, object/situation** | **Description (e.g., context, trigger)** |
| --- | --- | --- |
| *e.g., joy* | *patient* | *provider was joyous for the patient who could resume her sport after a long phase of absence due to her illness.* |
| … |  |  |

Emotion regulation (reported elsewhere)

Question 3: Whether and how did you regulate your emotions before, during, and after the selected interaction?

Question 4: What kind of emotional state did you want to attain by regulating your emotions?
